# A global dataset of publicly available dengue case count data

**DOI:** 10.1038/s41597-024-03120-7

**Published:** 2024-03-14

**Authors:** J. Clarke, A. Lim, P. Gupte, D. M. Pigott, W. G. van Panhuis, O. J. Brady

**Affiliations:** 1 Department of Infectious Disease Epidemiology and Dynamics, London School of Hygiene and Tropical Medicine, London, WC1E 7HT UK; 2 Centre for the Mathematical Modelling of Infectious Diseases, London School of Hygiene and Tropical Medicine, London, WC1E 7HT UK; 3 Institute for Health Metrics and Evaluation, University of Washington, Seattle, WA USA; 4 Department of Health Metrics Sciences, School of Medicine, University of Washington, Seattle, WA USA; 5 National Institute of Allergy and Infectious Diseases, Bethesda, MD USA

**Keywords:** Viral infection, Epidemiology

## Abstract

OpenDengue is a global database of dengue case data collated from public sources and standardised and formatted to facilitate easy reanalysis. Dataset version 1.2 of this database contains information on over 56 million dengue cases from 102 countries between 1924 and 2023, making it the largest and most comprehensive dengue case database currently available. Over 95% of records are at the weekly or monthly temporal resolution and subnational data is available for 40 countries. To build OpenDengue we systematically searched databases, ministry of health websites, peer reviewed literature and Pro-MED mail reports and extracted denominator-based case count data. We undertake standardisation and error checking protocols to ensure consistency and resolve discrepancies. We meticulously documented the extraction process to ensure records are attributable and reproducible. The OpenDengue database remains under development with plans for further disaggregation and user contributions are encouraged. This new dataset can be used to better understand the long-term drivers of dengue transmission, improve estimates of disease burden, targeting and evaluation of interventions and improving future projections.

## Background & Summary

Dengue is an emerging infectious disease of global public health importance, with an estimated 100 million symptomatic infections per year^1^ in over 125 countries^2^. Dengue virus (DENV) is transmitted by *Aedes* mosquitoes and is responsible for the greatest burden of human viral disease transmitted by arthropod vectors, resulting in 10,000 deaths per year^2^. Environmental suitability for dengue transmission is expanding due to climate change, urbanisation and international travel^3^. It is predicted that 2.25 (1.27–2.80) billion more people will be at risk of dengue in 2080 compared to 2015, totalling 6.1 (4.7–6.9) billion, or over 60% of the world’s population^3^.

Tracking the expansion of the burden of dengue is challenging due to the difficulties in collecting and aggregating consistent and comparable dengue incidence and prevalence data. The most commonly available measure of dengue incidence consists of case data from passive surveillance^4^: cases are identified through people who are experiencing symptoms presenting to health care facilities, where clinical algorithms and/or laboratory diagnostics are used to diagnose individuals as a suspected, probable, or confirmed dengue case^5^. This case data is then subject to a variety of processing stages, typically within local/regional health departments and national Ministries of health (MoHs). Ministries of health publish aggregated dengue statistics to varying degrees of completeness in epidemiological bulletins, outbreak reports or disease dashboards.

While many countries regularly publish dengue case statistics, they can often be difficult to find and no single database aggregates data from multiple countries to assess trends at the global level. Gathering data across all dengue endemic regions would enable re-analysis to better understand the drivers of transmission, monitor progress towards disease reduction targets, evaluate the impact of public health interventions and model the possible future burden and spatial limits under different climate scenarios. The higher the spatial and temporal resolution of the data available, the more informative and locally-specific these analyses can be.

Several attempts to create regional and global databases for dengue case data exist, but each have encountered limitations (Table 1). For decades, the World Health Organization (WHO) has received aggregated reporting of dengue by country level, once or twice a year or when outbreaks are occurring which is not timely enough to update for detailed analysis of dengue incidence and spread. Project Tycho covers 80 countries from 1960–2012^6^ with machine readable downloads, but has not been updated past 2012 and provides data for only two countries at administrative level 2 (Admin2) spatial resolution and none at weekly resolution.

**Table 1:** Comparison of other dengue databases and OpenDengue.

Source Category	Temporal		Spatial		Disease				Person	
	Resolution	Coverage	Resolution	Coverage	Severity	Serotype	Lab diag.	Mortality	Age	Gender
**OpenDengue**	W,M,Y	1924–2023	Country/A1/A2	Global (102 countries)	No	No	No	No	No	No
**Tycho**	M,Y	1960–2012	National/A1/(A2)	Global (80 countries)	Yes	No	No	No	No	No
**PAHO PLISA**	W	2014-current (2024)	National/A1	Americas (56 countries)	Yes	Yes	Yes	Yes	No	No
**ECDC**	Y	2008-current (2024)	National	Europe	No	No	No	Yes	Yes	Yes
**GIDEON**	W,M,Y*	1780-current (2024)	National/subnational*	Global	Partial*					
**Pro-MED mail**	W,M,Y*	1996-current (2024)	National/subnational*	Global	Partial*					

*Data availability varies by source and may contain spatially or temporally non-continuous records. M/W/Y = Monthly/Weekly/Yearly. A1/A2 = 1^st^/2^nd^ national administrative unit resolution. Lab diag. = information of laboratory diagnostic method used.

The Pan American Health Organization (PAHO) Health Information Platform for the Americas Database (PLISA)^7^ is a comprehensive and user-friendly resource with weekly data on dengue cases with extensive metadata, though it does not provide global coverage. In this repository, only 9 countries publicly report subnational data, there are data gaps and the focus is on cumulative case counts (as opposed to more informative incidence). Efforts to aggregate data from other WHO regions into a unified platform (DengueNet^8^ then later Dengue Explorer^9^) have struggled with consistency of reporting, contemporariness and are only available at a national level^10^. The European Centre for Disease Control (ECDC) has a surveillance atlas of infectious disease^11^ from 2011–2021, though it is for European (non dengue-endemic) countries only at annual resolution. The Global Burden of Disease Project^12^ collects and makes publicly available national estimates of dengue incidence 1990–2019 and the original source locations of their data can be viewed in using the GHDx platform (https://ghdx.healthdata.org), but tables of the extracted data are not publicly available. GIDEON^13^ is an infectious disease database that is regularly updated with outbreak reports and has a dengue dashboard for the majority of dengue affected countries, but is a paid for subscription service. ProMED mail^14^ collects reports globally and reports them daily by region, but does not have tabular machine readable download options and while a very useful resource, requires substantial manual processing.

To date, no repository has been able to combine global coverage, public availability, machine readable accessible formats at high spatial-temporal resolution and sustained updates over long time periods. However, two recent developments have made this task more feasible for dengue. First, there has been a gradual but extensive global investment in digital data collection and analysis for health surveillance worldwide utilising systems like DHIS2 (https://dhis2.org). This has increased the coverage, speed, reliability and accessibility of surveillance data, particularly for infectious diseases. Second, the COVID-19 pandemic has shown the demand for making infectious disease data publicly available and the value platforms to display and re-analyse such data can add to the epidemic response^15^. These trends are increasingly internationally recognised with a central aim of the World Health Organization Global Arbovirus Initiative being the development of better real-time data analytics at the global level^16^.

**Fig. 1:**
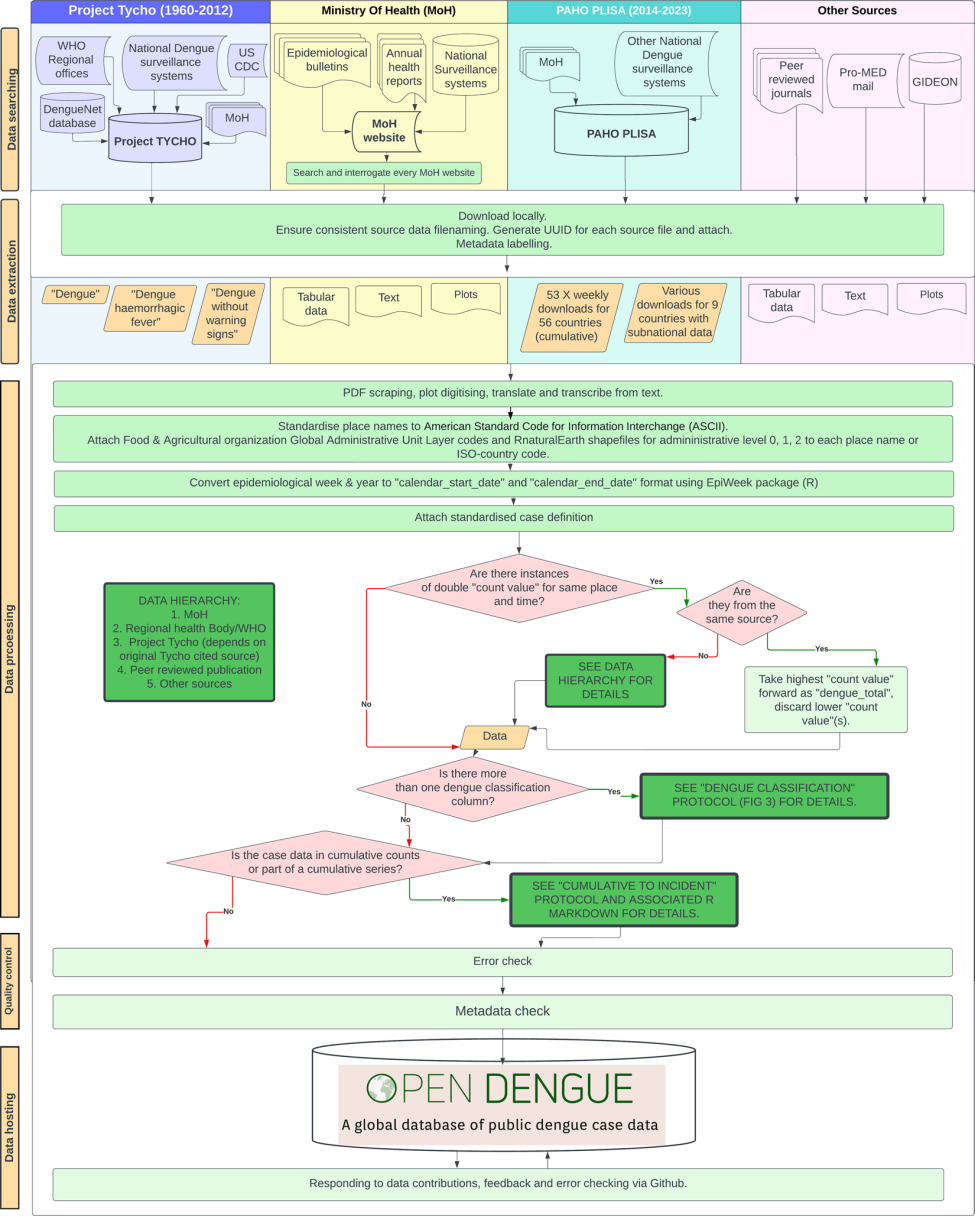
Detailed schematic of OpenDengue methods through data searching, extraction, processing, quality control and hosting. Columns have coloured backgrounds according to the original source category, including Project Tycho, Ministry of Health, PAHO PLISA and other sources. Coloured columns contain methods applied only to that specific source. White background contains methods applied to data from all source categories. Emboldened green boxes lead to more detailed protocols included in this publication. Red diamonds represent decision trees. Light green rectangles represent standardised processes. Orange rhombus represents data. Figure source: OpenDengue.org.

Dengue case data exists in multiple formats from a wide variety of sources that require various processing methodologies^17^. Detailed source metadata is important to ensure case counts can be traced back to their original reporting source, and to enable assessment of comparability between sources. Locating, extracting, processing and standardising data all takes time but is essential to enable reuse and re-analysis^17^. Here we describe our efforts to search, extract and format publicly reported dengue timeseries, population-level case data at the highest spatial and temporal resolution from across the dengue endemic world (Fig. 1). We also describe how this data is packaged into a publicly available database and website that promotes re-use.

## Methods

### Search Strategy

We searched four main source categories for dengue data: MoH websites, existing infectious disease databases, peer-reviewed journal publications, and ProMED mail^14^ (Fig. 1). Through an initial comparison of source categories based on factors such as temporal and spatial resolution, contemporariness, geographical coverage, disaggregation by other variables and ability to download datasets in machine readable formats, we developed a source priority hierarchy to improve efficiency of data extraction and avoid duplicating aggregation efforts of others.

We began by searching existing aggregated databases (Project Tycho^6^), WHO regional databases (PAHO PLISA^7^, WPRO^18^) and national surveillance dashboards (e.g. Singapore^19^). WHO regional reports were searched for by each regional website, some of which were dengue specific, others found within multiple disease outbreak reports. Common sources of dengue data included epidemiological bulletins and annual health reports that were located using site maps and search options. Websites without English language options were navigated using Google translate and liaising with peers/colleagues from the country in question who developed regionally relevant search terms in the appropriate language. Peer-reviewed literature articles containing relevant data^20–33^ were searched for and located using Pubmed and cross references with country profiles on GIDEON^13^. ProMED mail was used for a small number of countries with high estimated burden and large data gaps. This required searching for the country name or region and time period in question. Search strategies became more targeted and effective as we developed more familiarity with each country’s reporting systems and methods of archiving of data. Data gaps were then evaluated once more after initial extraction and processing. Heatmaps of data coverage were regularly updated and used for targeted gap-filling based on estimated dengue burden, national and regional completeness.

Inclusion and exclusion criteria were developed to ensure consistency across source categories. The data source must be publicly accessible (e.g. on a ministry of health website) and denominator based (e.g. 10 cases from a defined population over a defined period of time). Case definitions vary by country and can include but are not limited to “suspected”, “probable”, “laboratory confirmed”. Cases can be disaggregated by severity based on either the 1997 (dengue fever, dengue haemorrhagic fever or dengue shock syndrome) or 2009 (dengue with/without warning signs, severe dengue) WHO case definitions^34^. At this searching stage, we included all levels of disaggregation, case definitions and disease severity. Specific case definitions as reported are included in the data record where they were clearly stated at source. We excluded imported cases where they were reported distinctly from autochthonous cases. Data not attributed to government public surveillance systems, e.g. reports on dengue that are available online through searches of the grey literature but are not linked back to the original source, were excluded. ProMED reports were included if they had a denominator or were associated with a place with clearly defined spatial limits (e.g. a city).

### Data extraction

While searching each source category, the data meeting the inclusion criteria were downloaded locally and onto shared cloud storage and saved by WHO region and by country (Fig. 1). Files were in various formats including “.csv”, “.XLS” and “.pdf”. Project Tycho^6^ was downloaded as.csv files for “dengue”, “dengue haemorrhagic fever” and “dengue without warning signs”. Ministry of Health data was downloaded as plots, tabular data and text. PAHO PLISA^7^ data was extracted as 53 weekly downloads of cumulative data for 56 countries with national level data. PAHO PLISA data was also extracted in multiple downloads for 9 countries with subnational data. “all cases” was the variable prioritised for each download in the PAHO PLISA dashboard. All character strings were converted to UTF-8 format. If many pdfs were downloaded for weekly epidemiological bulletins, these were combined into a single document spanning multiple weeks.

A standardised naming protocol was established to prevent duplicate source file names. A customised Universally Unique Identifier (UUID) for each source file was generated by using various components such as source categories (e.g., MoH, WHO, or Project Tycho), ISO3 country codes (or area names if applicable), time periods, and serial numbers (e.g., “MOH-MEX-2012-Y01-01”). An UUID is assigned to each dengue case count data record so that users can identify the source file by referencing source data (see section “Source data”).

If the data were provided in a table, it was scraped using PDF scraping R packages (*pdftools*^35^ and *tabulizer*^36^) or Microsoft Excel^37^. The tables were kept in their original format but only relevant columns were extracted. If the data were provided in a figure, it was extracted using WebPlotDigitzer^38^. If the data were provided in text, this was translated by Google translate where required and transcribed into tabular format by hand. If the data was in a map, where possible, individual case counts were extracted manually. If data were grouped into categories (eg. 1–10 cases, 11–100 cases), this data was not extracted. All extracted data files were saved in.csv format and processed (transformation or aggregation) separately using R if necessary. These processed files were then standardised to ensure they shared the same column names before being merged into one consolidated.csv file.

### Source data

Source data relevant to the original data source category that was searched, located and extracted was stored in a corresponding version-specific “.csv” file. Information such as date accessed, URL of main landing page, steps taken through website/sitemap navigation, relevant search terms used, and other relevant notes such as positioning on page are included. The data source can be identified and interrogated by looking up the record UUID in the sourcedata_V1.2.csv file then checking the corresponding URL or the archived download of the source in the repository^39^.

Case definitions for all data points have been extracted and standardised into three levels: suspected, probable, and confirmed, with the original wording included in source data. The specific case definition used for a particular data point will depend on the original data source. Typical descriptors at source include “confirmed”, “probable”, “suspected”, “total” or simply “dengue cases”. These may be included in text, axes labels, table or column headings. We extracted the corresponding case definition from each source file verbatim, using google translate where necessary. If the data source alone did not provide enough information to determine whether the cases reported were probable, suspected, or confirmed, we tried searching online for surveillance case definitions for different countries, visiting websites for the national surveillance system, and consulting national guidelines for dengue control. Where “dengue cases” or “total” was the only descriptor, and no further information available elsewhere, we adopted the case definition: “Report of all dengue cases; suspected, probable, confirmed, non-severe and severe cases, and deaths” following the international standard set by PAHO^7^.

### Metadata

Both the main OpenDengue data and the source data are accompanied by separate detailed “.json” format metadata files in the Figshare repository. These metadata files follow the National Institute of Allergy and Infectious Diseases (NIAID) Data Ecosystem Dataset schema (nde:dataset, https://discovery.biothings.io/ns/nde/nde:Dataset) which is based on the schema.org:dataset and bioschemas:dataset formats.

### Data Processing

Each record in the dataset corresponds to a dengue case count value for a non-overlapping unique location and time period. To identify overlapping data records, data records first went through standardised geomatching and time matching.

#### Geomatching

Records were matched to unique spatial entities based on the character description of the area. For convenience and flexibility we match data to two different internationally recognised shapefile formats: the United Nations Food and Agricultural Organization Global Administrative Unit Layers^40^ (FAO GAUL^41^, Admin0, Admin1 and Admin2) and the Natural Earth shapefiles (naturalearthdata.com downloaded via *rnaturalearth*^42^, Admin0 and Admin1). To improve character matching, text strings were capitalised, converted to American Standard Code for Information Interchange (ASCII) format and combined across admin unit levels e.g. “ARGENTINA, SALTA, ORAN”.

Country (Admin0) was matched to a unique three letter country code (ISO alpha-3 standard) using the *countrycode*^43^ R package. Sub-national administrative units were matched to GAUL codes (Admin1 and Admin2 in FAO GAUL) or ISO 3166-2 codes (Admin1 in Natural Earth) using hierarchical fuzzy matching (using *hmatch*^44^) that preserved Admin0-2 relationships. Non matching text strings were manually edited to the closest matching administrative unit based on text string correction or, where necessary, extraction of centroid latitude and longitude in Google Maps then cross referencing with the target shapefile. Brazil has its own designated geomatching package *geobr*^45^ which was used in combination with a FAO GAUL code lookup table^46^ to geomatch

Brazilian Instituto Brasileiro de Geografia e Estatística (IBGE) codes to GAUL codes at the admin 2 level.

#### Time matching

Date records were converted to calendar year time formats. While some data sources were already in calendar date format, some reported cases using “epidemiological weeks”. We converted this format to calendar start and calendar end date using the EpiWeek^47^ package in R^48^. This function defines the first epidemiological week of the year as containing at least four days in January and the first day of each epidemiological week starts on a Sunday and ends on a Saturday. This is in line with the US CDC version of epidemiological week^47^. Cumulative data underwent additional time matching processing (see section “Cumulative to incident case count conversion in the PAHO PLISA database”).

#### Conflicting records

After geomatching and time matching, we were able to search for instances where multiple different sources report different case counts for the same location and time period. We call these “double count” values. If these were exact duplicates of the same dengue case count for the same place and time, the duplicate was discarded. If the dengue case counts were different, or conflicting, for the same place and time, we followed our double count protocol and data hierarchy (Fig. 1). If they were from the same source category, we took the highest count value forward as our dengue total and discarded the lower value(s). If they were from different sources, source categories were prioritised in the following order (highest priority to lowest): Ministry of Health report, regional health body report, Project Tycho, peer reviewed journal publications, opportunistic sources. In cases where there are multiple sources other than the Ministry of Health (e.g., WHO versus Project Tycho) reporting different numbers of dengue cases, the original source names of Tycho were interrogated (available from the source file) and records from Tycho were taken only if they were from MoH.

Because some conflicting records contained superior spatial or temporal resolution data, three extracts of the OpenDengue database are available for download by users: i) the best estimate of total national (Admin0) case counts, ii) maximised temporal disaggregation and iii) maximised spatial disaggregation. For each of these, records that report the highest case count, highest resolution temporal counts and highest spatial resolution counts, respectively, are prioritised where conflicting records exist. It should be noted that we did not alter records to be spatially consistent, e.g. the sum of all cases in a particular country at Admin2 level may not match total cases reported at Admin0 level even if over the same time period. This decision was made to preserve consistency with the original sources.

#### Dengue classification

The OpenDengue version 1.2 dataset contains reported total case counts with each row corresponding to a unique location and time. Where reported, we include dengue cases at all levels of severity (dengue, dengue with/without warning signs, severe dengue, dengue haemorrhagic fever, dengue shock syndrome, dengue deaths) and methods of confirmation (suspected, probable, clinically confirmed, laboratory confirmed) in the variable “dengue_total”. The corresponding case definition is included in the variable “case_definition_standardised”.

**Fig. 2:**
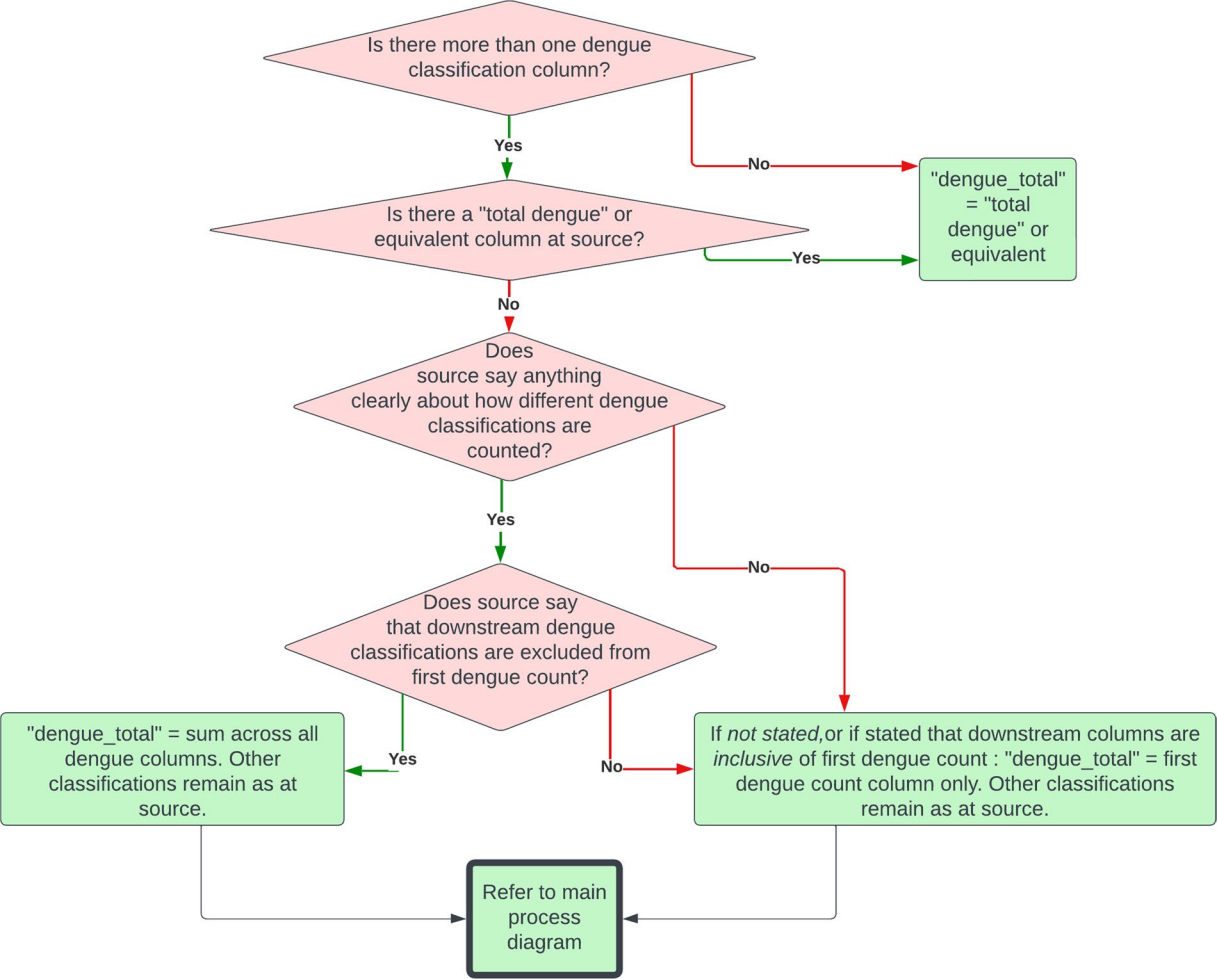
Dengue classification protocol used to determine total dengue case counts in OpenDengue when there was more than one “dengue” or equivalent column at source. Red diamonds represent decision trees, green shaded rectangles data processing stages. See Fig. 1 for context of this protocol within the overall OpenDengue methodology. Figure source: OpenDengue.org.

There are different classifications of severity of dengue which change by place and over time^34^. Different source categories report the case counts with varying levels of disaggregation by disease severity and methods of confirmation. Some sources disaggregate dengue cases by severity or other attributes that may or may not be mutually exclusive, making the total number of dengue cases reported unclear. To resolve this, we followed our dengue classification protocol and systematically measured total cases (Fig. 2). Downstream dengue classifications correspond to possible sequelae of dengue infection; “severe dengue”, “dengue haemorrhagic fever” or “deaths”.

#### Cumulative to incident case count conversion in the PAHO PLISA database

The PAHO PLISA platform allows downloads of dengue case count information at a variety of spatial and temporal scales with different case definitions that are not necessarily directly comparable. PAHO only reports cumulative case datasets for “all dengue cases: suspected, probable, confirmed, non-severe and severe cases, and deaths.” Records in this dataset are in incidence format (as opposed to cumulative incidence) because incidence has a temporally fixed denominator e.g. cases 23-29th January as opposed to cases by 29th January and therefore avoids ambiguity over when cases occurred when records are subject to repeated revision (as is common in the PAHO PLISA data).

**Fig. 3:**
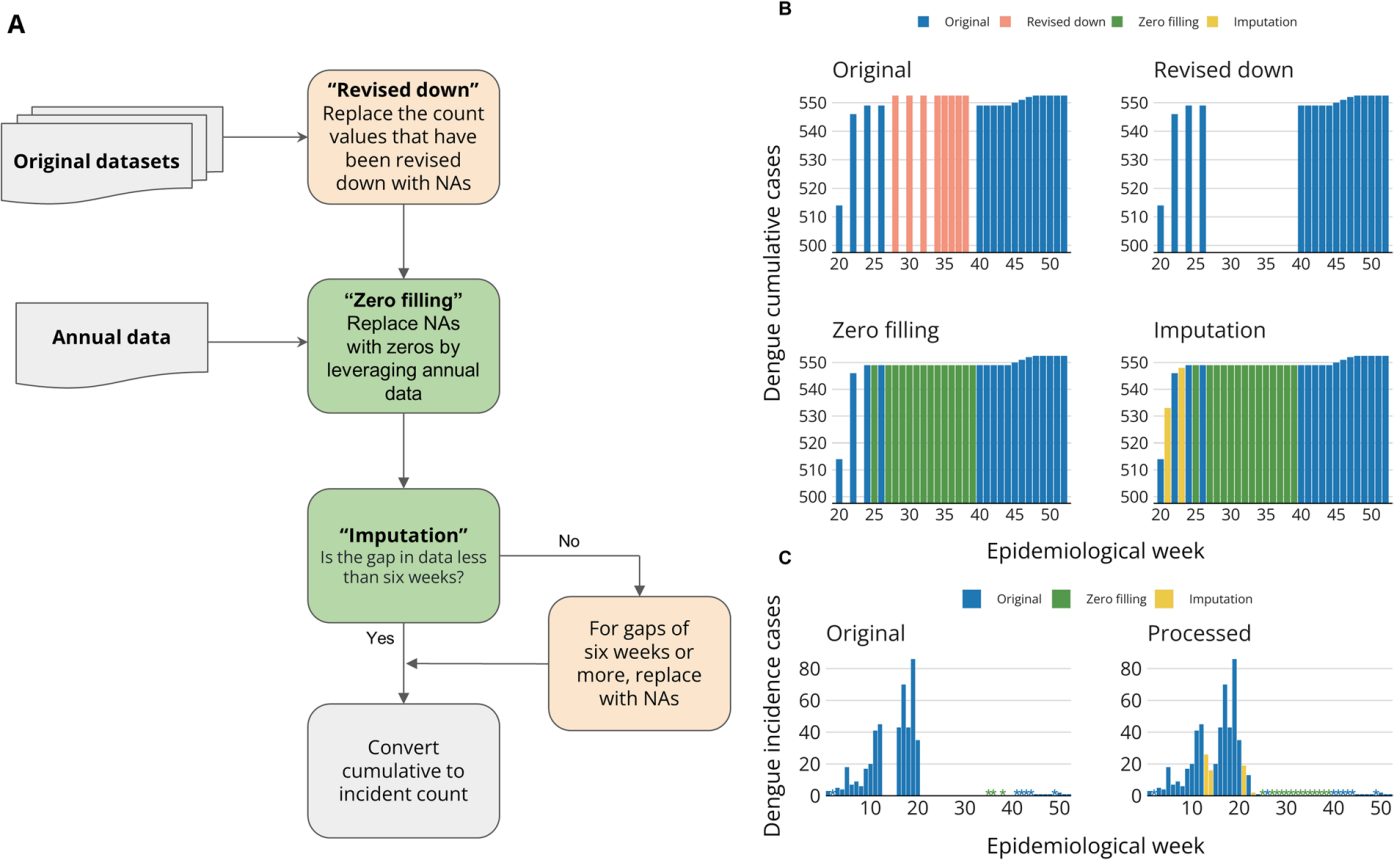
(**A**) Data processing flow chart for PAHO PLISA data; (**B**) an example of each stage of the data processing using Argentina 2017 data. Note the original cumulative values in orange for weeks 27–38 are greater than the following week 40, which has been revised down, leading to negative incident counts if included. These are replaced with NA. (**C**) comparison of incident time series between original and final processed data for Argentina 2017. Asterisks in the bar chart indicate zero dengue cases. Figure source: OpenDengue.org.

The cumulative PAHO PLISA data poses four main challenges that we have worked to resolve. There are frequently large increases in particular weeks of cumulative counts following flat cases or reports of absence. It is unclear if these jumps represent a sudden increase in dengue transmission or heaped reporting. There is revising down of cumulative values over time, as reports mature from revised to finalised status. These would result in negative incident counts for some weeks. There are gaps in cumulative reporting, where it is unclear what the incident counts would be for the intervening weeks. Some countries report no cumulative data, where it is unclear if this is a record of absence of dengue or an absence in reporting. Our solutions to solve each of these issues are detailed in this section and a Rmarkdown file in the OpenDengue Github repository^49^ provides a detailed step-by-step walkthrough. They include dealing with revising down of data, “zero filling” and imputation (Fig. 3). A small minority of total data records in OpenDengue version 1.2 have gone through these processes (see section “Technical Validation”), meaning impact on regional or national trends are minimal, but may have an influence for analyses of specific time periods. Records that have been processed with these steps can be identified in the dataset by the addition of the suffix “(Zero filling)”, “(Imputed)” to the UUID variable. Users who wish to use alternative methods to impute or zero-fill data can use this identifier to remove these records and implement their own gap filling algorithms of choice. PAHO PLISA portal only permits download of all countries cumulative data sets in a week by week fashion, by moving the epidemiological week slider while selecting all countries. The cumulative dataset has “select epidemiological week” slider options for all available epidemiological weeks of every year. However, the epidemiological week for which information is available/reported can differ from that on the slider. Many countries have missing weeks of data. The extent of missing data varies greatly between countries, and over time. We also downloaded overall national, annual counts for each country (Fig. 3A).

The weekly downloads require the file format to be encoded differently and re-saved in csv format for further processing. The data then undergoes geomatching and time-matching as per the above methods. Raw cumulative case count data is available from the source file. Here, the calendar start date is fixed at the beginning of epidemiological week one of each year, and the calendar end date is moved forward to match the corresponding week of the respective cumulative case count report. When the cumulative count for a time period was lower than for the preceding period, we considered that count to be unreliable as it resulted in a negative incident count, and replaced it with NA (missing value). 44 of the 52 countries in the Americas had values which were revised down and replaced with NAs (Fig. 3B).

We downloaded annual, national level case counts from the same PAHO PLISA source. We considered these annual counts to be the most correct mature annual-level summary of the data. We proceeded to replace NAs with zero incident cases by leveraging these annual counts, a process we call “zero filling” (Fig. 3B). With data still in cumulative format, this “zero filling” leads to a flattening of the cumulative case counts as they stay constant.

We performed “zero filling” for three specific record gap scenarios (Fig. 3B). Scenario 1 is where we have an entire year without any case reporting from the cumulative weekly dataset, and a zero annual count. Here, we imputed zeros for all other epidemiological weeks in the year. Scenario 2 is where there is missing data in the cumulative weekly dataset, and the final cumulative weekly total is equal to the annual total. Here, we imputed zeros for all weeks after the last cumulative weekly count. Scenario 3 is where duplicated cumulative counts in the weekly dataset have missing valuess in between them.

To further support our conversion from cumulative to incident case counts, we imputed gaps of less than six weeks in the cumulative dataset that had undergone NA replacement for revised down values and “zero filling”. We chose six weeks or less as the threshold suitable for imputation to preserve the temporal continuity of the dataset to limit the introduction of artificial trends or inaccuracies. We used the *Zoo* R package^50^. We performed cubic spline temporal interpolation via the “na.spline” function. We inspected the imputed incident time series for comparison (Fig. 3C).

#### The OpenDengue.org website

To facilitate open and efficient access to the OpenDengue database, we developed a dedicated website (opendengue.org) using R Markdown and GitHub Pages. Aside from providing comprehensive access to the database (and dataset version 1.2- described in this article) via our Git repository, the website provides a user-friendly web-based application to visualise heatmaps showing data coverage and time series data for specific times and regions through customisable interfaces using *Shiny* and *Plotly*^51^. The website and associated GitHub repository also encourages user submissions to fill data gaps via the GitHub issues tracker which has already facilitated the identification of additional data sources with sizeable gaps filled in Bhutan and Taiwan.

## Data Records

The latest dataset (currently version 1.2) is available on our OpenDengue website (https://opendengue.org/data.html). Past and current versions are also available in the OpenDengue Github repository (https://github.com/OpenDengue/master-repo). Dataset version 1.2 is the version of the database that has been peer reviewed and is described in this article. Files for the main case dataset and source data have been deposited in the cited Figshare repository in csv format^39^. All data and metadata in OpenDengue conforms to FAIR standards^52^. To provide flexibility to users, we have geomatched each dengue case count data entry to both FAO GAUL codes^40^ and RnaturalEarth^53^ shapefile codes.

Different data types were available at higher spatial or temporal resolutions. For example, a source category may have national level data available at a weekly resolution, and sub-national level data available at monthly or annual resolution only. To resolve this, we provide three global summaries of the data in OpenDengue. We provide the best national estimate, the best temporal resolution and the best spatial resolution. This allows users to customise their data extraction based on their research question.

Each row in the data table contains a unique, non-overlapping location and time period with the associated dengue case data. The below codebook describes each variable:

adm_0_name: administrative level 0/country name

adm_1_name: administrative level 1 name

adm_2_name: administrative level 2 name

full_name: full place name ISO_A0: ISO country code

FAO_GAUL_code: Food and Agricultural Organization Global Administrative Unit Layer Code

RNE_iso_code: RnaturalEarth ISO code

IBGE_code: Brazilian Instituto Brasileiro de Geografia e Estatística (IBGE) code

calendar_start_date: the start date in calendar time with the format YYYY-mm-dd

calendar_end_date: the end date in calendar time with the format YYYY-mm-dd

Year: Year

Dengue_total: the total dengue case count relating to the period and place (see sections “Dengue classification” and “Conflicting Records”)

case_definition_standardised: case definition after standardisation

S_res: spatial resolution

T_res: temporal resolution

UUID: Universal Unique Identifier relating to the source file from which the data originates

### Data summary

**Fig. 4:**
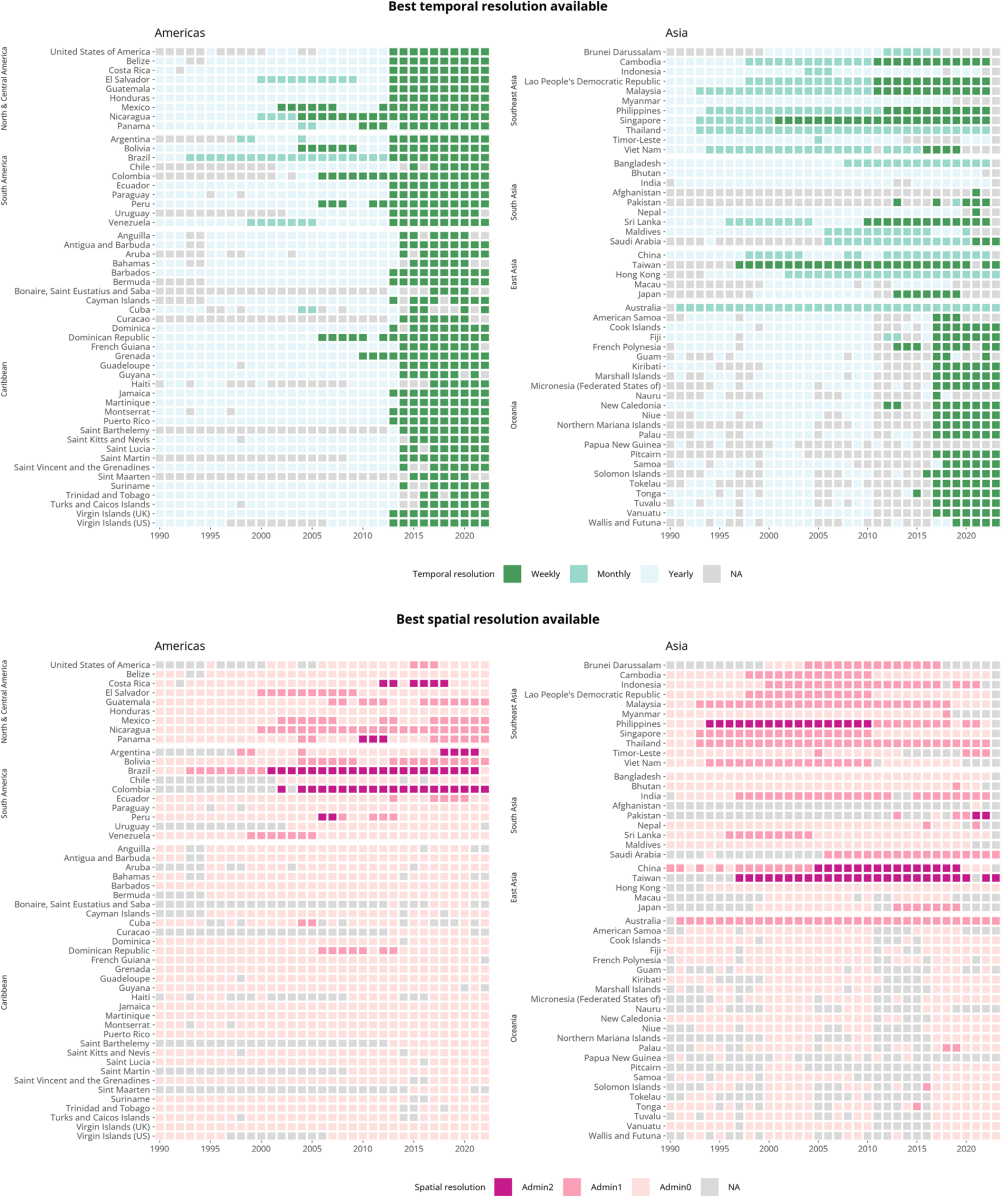
Heatmaps showing the best spatial and temporal resolution available of dengue case data in current version of OpenDengue 1.2. Interactive version available at OpenDengue webpage. Figure source: OpenDengue.org.

Version 1.2 of the OpenDengue dataset includes information on over 56 million dengue cases distributed over 102 countries for the time period 1924–2023. We combine data from 843 different sources with 99.8% of the data records being at weekly or monthly temporal resolution and sub-national data is available for 40 countries. Heatmaps showing data coverage are shown in Fig. 4 with interactive versions available on the OpenDengue website. These show good coverage across all dengue endemic regions with general improvements in completeness and temporal resolution over time. Priority areas for future data collection include: Data for Pacific Island nations over the period 2011–2016, more weekly resolution data for recent time periods in Asia to bring records in line with those in the Americas and greater subnational disaggregation of data from South Asia (India, Bangladesh, Nepal and Pakistan).

**Fig. 5:**
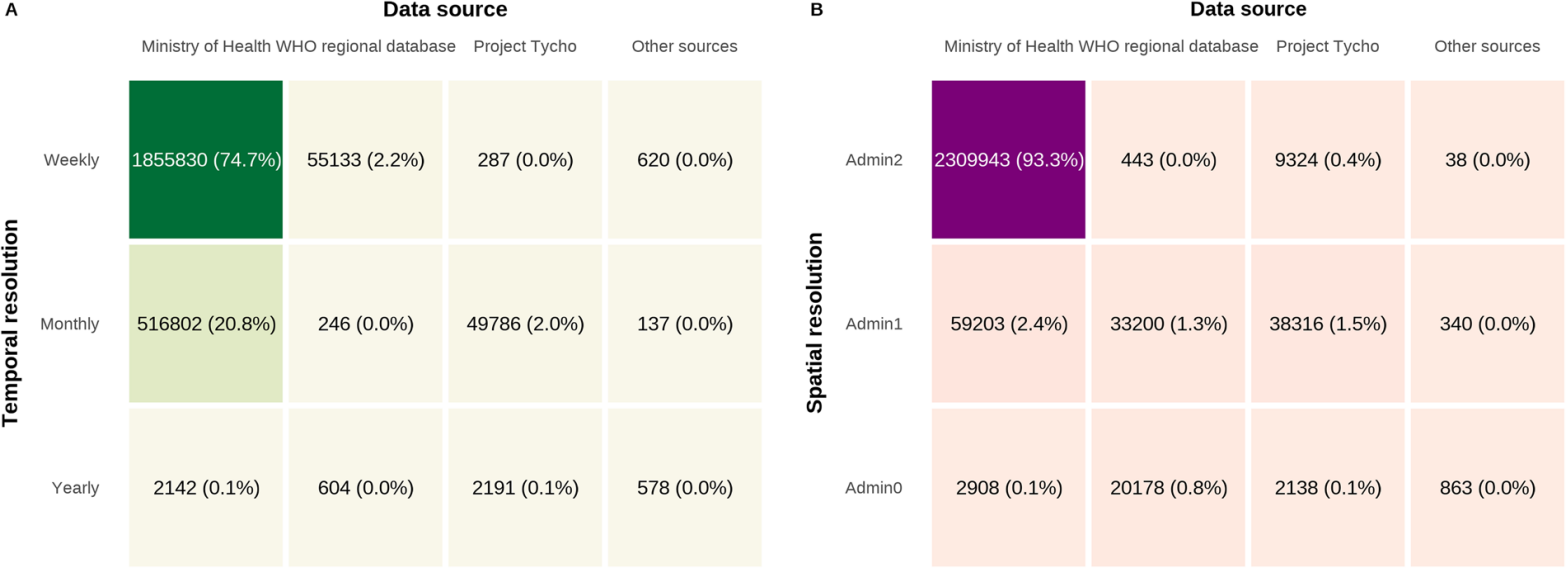
Data source categories contributions to OpenDengue 1.2. Percentages in brackets indicate the percentage of the total dataset that is available at that temporal or spatial resolution and from that data source category.

The majority (>95%) of our data records were obtained from ministry of health sources (Fig. 5). The largest contributors to this high percentage were ministries of health from Brazil, Colombia, the Philippines, China and Taiwan who all report weekly case counts at the second administrative level and usually provide such data in machine readable formats via online databases. The OpenDengue database presents a substantial advance over the existing WHO regional databases or Project Tycho, containing approximately 50 times the data records by pooling data from a variety of sources. While data from “Other sources” made up a proportionally negligible contribution overall (Fig. 5) they were essential in filling key spatial and temporal gaps in the database (Fig. 4) to ensure geographic and temporal completeness.

**Fig. 6:**
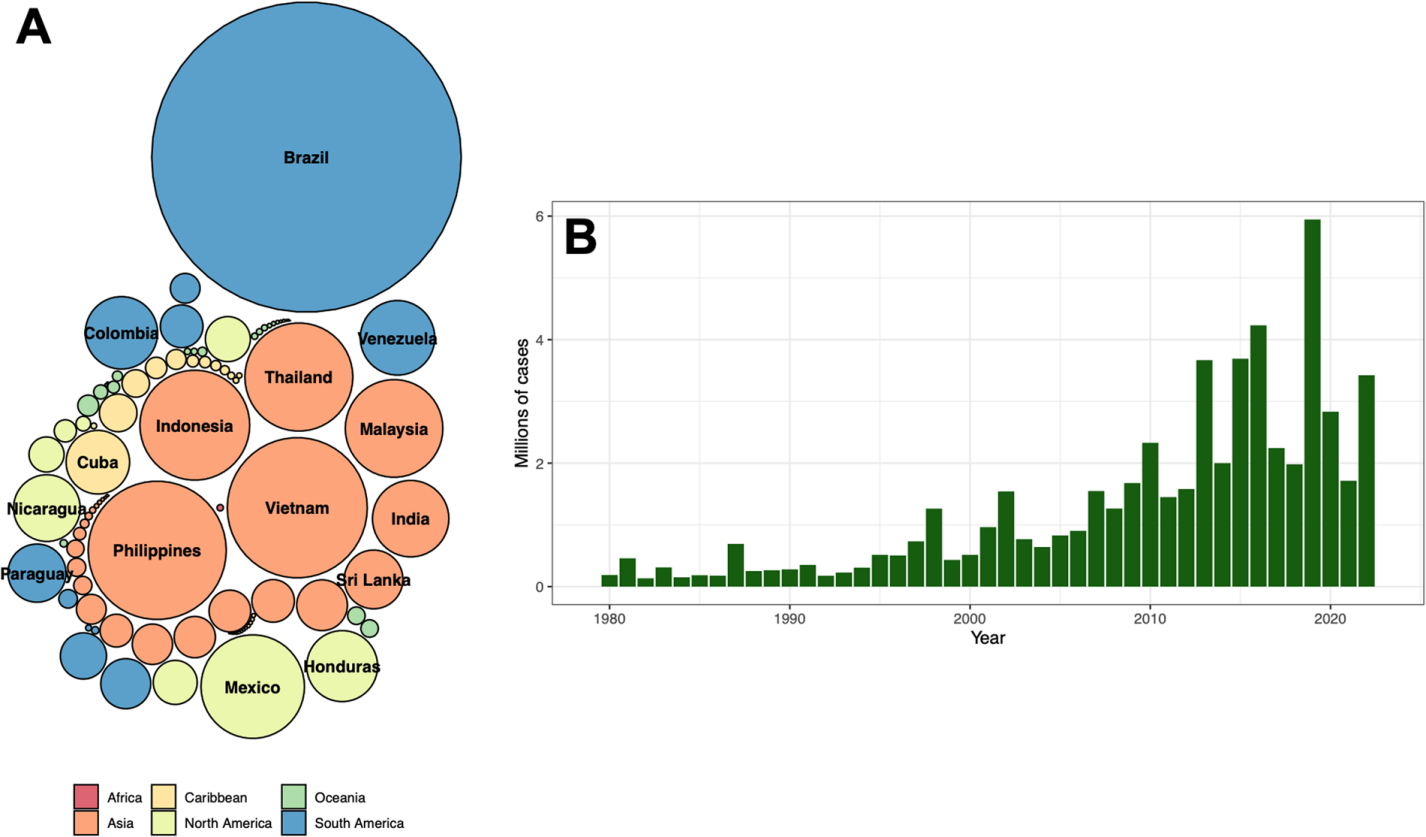
The total number of reported dengue cases in each country (**A** circle area proportional to total cases, top 15 countries labelled) and year (**B**, only counts from 1980 onwards shown).

When analysing the total number of cases reported in OpenDengue version 1.2, the most cases were reported from Brazil (22.0 m), followed by Vietnam (4.5 m), the Philippines (4.4 m) and Indonesia (2.8 m, Fig. 6A). A total of 34 countries reported more than 100,000 cases over the time period, showing that OpenDengue can be used for reanalysis across many different high burden countries. When examining trends over time (Fig. 6B), the number of reported cases has risen substantially over time with particular increases since 2008 with over 1 million cases reported every year since.

## Technical Validation

### Cumulative to incident data technical validation

**Fig. 7:**
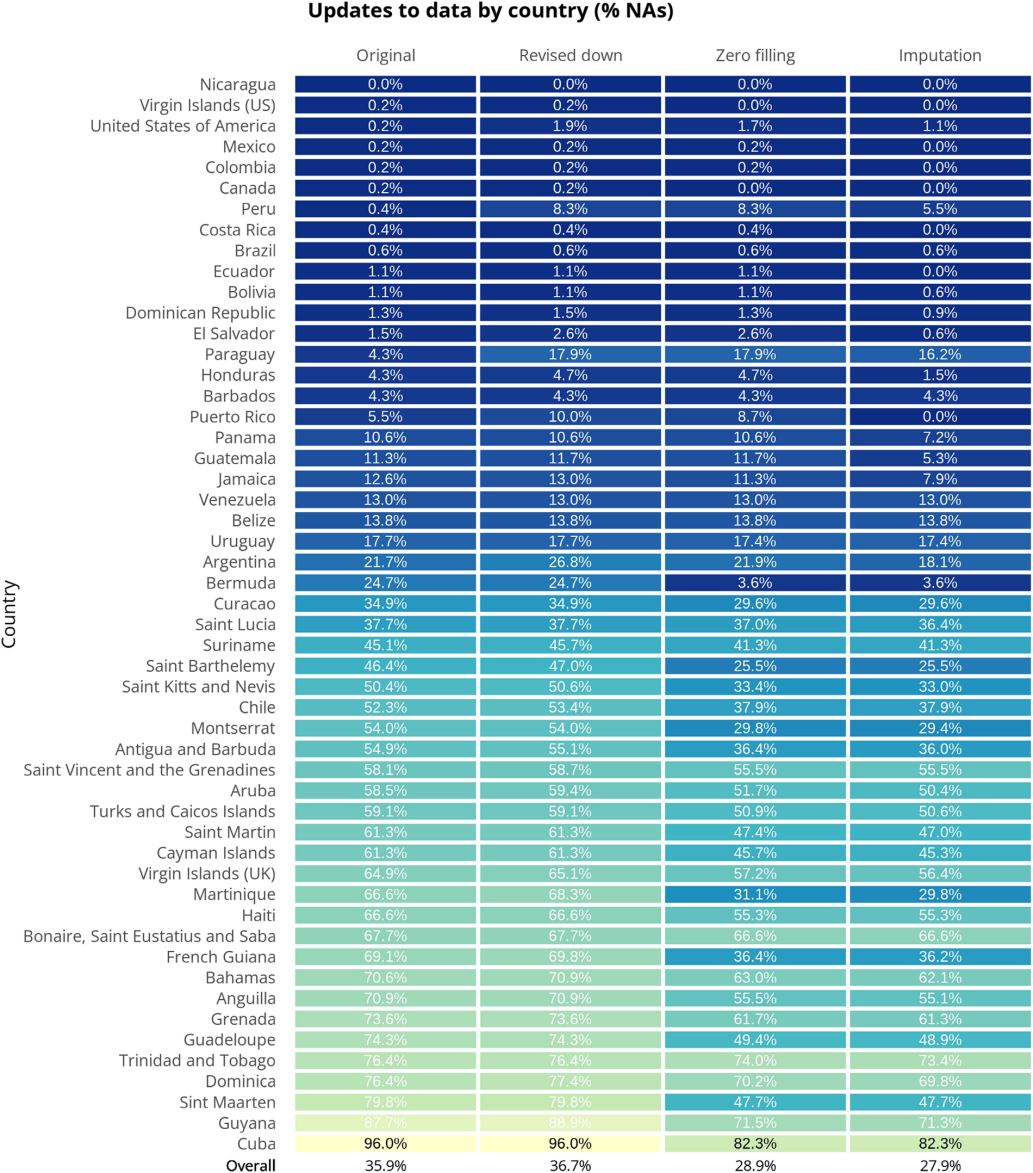
A summary of the improvement in completeness to the original raw PAHO PLISA data by country that each stage of processing (revise down, zero filling and imputation) has contributed. The figures refer to the percentage of data between 2014–2022 that is not available (NA). A reduction in this figure (i.e. colour change from yellow to blue) when compared to the first column represents an increase in completeness for the respective country.

Multiple technical validation stages were built into our cumulative to incident data strategy for PAHO PLISA. We compared total weekly case counts over the year with annual counts to validate they were equivalent, any discrepancies were investigated and resolved using our data hierarchy or conflicting records protocol. We inspected cumulative time-series data for all countries visually (Fig. 3C). “Zero filling” was successful in filling substantial data gaps for 36 of the 52 countries in the Americas. We reduced the overall percentage of missing data (NA) from 35.9% to 28.9% with this method (Fig. 7). We performed imputation for 34 countries where records met our criteria for imputation. Imputation filled a much smaller proportion of each country’s missing values, with the maximum being 8.7% for Puerto Rico and the overall reduction in missing data being 1% (Fig. 7). Of 24,440 rows, 151 rows have been replaced with NAs following the ‘Revised down’ stage. In total 2,155 gaps have been filled following the ‘Zero filling’ and ‘Imputation’ stage.

### Database-wide technical validation

After collating time series data for all countries, JC, AL, and OJB independently reviewed it, assessing time series plots visually to check for obviously unusual disease trends (e.g. anomalous spikes in case counts) or errors in calendar-time matching. We also compared these plots with available incident plots from regional reports from which they were extracted or PAHO PLISA incident plots. Notably, the PAHO PLISA incident plots are non-severe cases only, whereas OpenDengue is all cases, but they remained helpful to indicate overall trends are aligned between OpenDengue and PAHO PLISA after processing. We validated our source data table through the generation of UUID for each source file. We systematically cross-referenced all UUIDS in the database with their corresponding UUID in the source data for omissions or errors. Duplicates or double count values were checked using our double count protocol and data hierarchy. An error in this protocol led to OpenDengue 1.1 having some duplicate counts for the Americas. This was remedied for version 1.2. Complications arising from differing dengue classifications were systematically checked by our dengue classification protocol (Fig. 2).

Finally, we performed regular random data checks on opendengue, ensuring that the row spotlighted for checking could be accurately traced back to a source document and the case counts were correct. The first version (version 1.0) of this dataset has also been publicly available since June 2023 and we encourage anyone to test the data and raise any errors or contributions via the issues tab in our GitHub repository.

## Usage Notes

OpenDengue draws together and standardises data from multiple sources that enable new analyses at global and regional scales. Examples include identifying worst affected areas and years, understanding drivers of transmission such as climate factors and interventions and predicting future trends and outbreak risk.

### Choice of extract

Users should consider which data extraction (national, spatial or temporal) is most relevant for their research question (see section “Data Records”). Applications that explore changes in dengue dynamics over time including time series analysis, forecasting and national programme evaluation should use the temporal extract which preferentially selects data records that maximise the temporal resolution of the data. Analyses that focus on specific sub-national locations or geospatial analyses should use the spatial OpenDengue extract where spatial resolution of the data is maximised. The national extract provides the single best (highest) estimate of annual cases for each country, regardless of the spatial or temporal scale of the original sources and is thus best suited for burden estimation and broader scale analyses that explore national-level determinants of longer-term dengue trends. Each of these three extracts have a high degree of overlap in sources and case counts, but there are specific settings where exact choice may be important.

### Limitations on comparability

As with all disease surveillance databases there are several important limitations to consider regarding the accuracy of dengue data collection and reporting and biases affecting each stage of the system, with numerous proposals for refinement. Like many diseases, dengue surveillance, reporting and accessibility of data can vary substantially between and within countries, reporting sources and over time which may affect comparability of this data. While the global coverage and 30+ year coverage of OpenDengue is of considerable benefit to users, we encourage caution when making comparisons between countries, or within countries over time. Some insights into comparability can be gained from using the OpenDengue standardised case definition variable. Records that report “confirmed” cases are likely to be less sensitive but more specific than records that use “probable” or “suspected” cases. However, even within these standardised case definitions there are a broad range of different national case definitions that may vary, particularly around the transition from case definitions based on the WHO 1997^54^ and 2009^55^ criteria, which in some cases take effect long after 1997 or 2009 respectively. We encourage users to use the OpenDengue source data to examine the original source and the details of its chosen case definition to assess comparability. All case definitions excluded imported cases when they were reported distinct from autochthonous cases, but methods of distinguishing imported from local cases vary. Data from some areas, particularly at the northern and southern limits of transmission, may contain a mixture of imported and autochthonous cases, and this is the reason why we did not systematically include and separate imported and autochthonous cases. Users interested in analysing imported case data should investigate if the databases collated by the USA^56^ and ECDC^57^ or the Geosentinnel network (geosentinel.org) may better fit their aims. For clarity, it is also important to state that even in countries with established surveillance systems, reported cases make up a small but variable fraction of total dengue infections due to asymptomatic infection, heterogeneities in treatment seeking rates, treatment in the private sector and challenges of accurate diagnosis in primary healthcare settings.

For detailed analysis on certain countries or regions, we encourage users to get in touch with local experts or health agencies. Such interaction can be helpful to better understand the process of generating the reported data. This can be useful to check if specific observed patterns or inferred drivers are actually a result of changes in case definition or reporting practices over time or between areas.

### Dataset version control

The OpenDengue database is under continual development with periodic new version releases. We aim to release new versions of the dataset at least every six months with new versions deposited in the same Figshare repository with different DOIs. It is recommended for users to specify which version of OpenDengue they use in their analyses and routinely check for updates at relevant points in their project lifecycle. The content of this article is relevant for OpenDengue version 1.2 but all current and past versions of the dataset are available in the OpenDengue Github repository^49^. Future versions of the dataset will include additional data (either addressing gaps or improving spatial and temporal resolution) with plans to disaggregate dengue case data by severity, method of confirmation, age and serotype where possible.

### Citation and data licence

OpenDengue data is made available under a creative commons CC BY-SA licence. This allows all potential users (commercial and non commercial) to reuse and adapt the dataset with appropriate acknowledgement. Under a CC BY-SA licence all adaptations of the OpenDengue dataset must also be made available under the same terms. The preferred citation for OpenDengue is citation of this manuscript in addition to the Figshare repository link to the specific version of the dataset used:

“Clarke, Joe; Lim, Ahyoung; Gupte, Pratik R.; Pigott, David M.; van Panhuis, Wilbert G; Brady, Oliver (2023). OpenDengue: data from the OpenDengue database. Version [1.2]. figshare. Dataset^58^. 10.6084/m9.figshare.24259573”

Where possible, we encourage users to also cite the original sources of the data which can be identified from the source data file using the record UUID.

### Contributing data to OpenDengue and feedback

While we have aimed to be as comprehensive as possible in our searches for publicly-available dengue data, additional sources will inevitably become available. If users are aware of dengue data from places and times where there are gaps in our database, contributions are very much encouraged. A dedicated page on the OpenDengue website (https://opendengue.org/contribute) details how users can notify us of additional records and the information that is useful to provide. With their permission, contributors will be acknowledged in the source data and brief news items disseminated via social media for larger data contributions. Similarly, we also welcome user contributions to identify possible errors in the database or general feedback on the formatting which will be considered and addressed where possible.

## Data Availability

All code used to process and standardise the data are included in the OpenDengue Github repository^49^.
